# Anti-Ephrin Type-B Receptor 2 (EphB2) and Anti-Three Prime Histone mRNA EXonuclease 1 (THEX1) Autoantibodies in Scleroderma and Lupus

**DOI:** 10.1371/journal.pone.0160283

**Published:** 2016-09-12

**Authors:** Doua F. Azzouz, Gabriel V. Martin, Fanny Arnoux, Nathalie Balandraud, Thierry Martin, Sylvain Dubucquoi, Eric Hachulla, Dominique Farge-Bancel, Kiet Tiev, Jean Cabane, Nathalie Bardin, Laurent Chiche, Marielle Martin, Eléonore C. Caillet, Sami B. Kanaan, Jean Robert Harlé, Brigitte Granel, Elisabeth Diot, Jean Roudier, Isabelle Auger, Nathalie C. Lambert

**Affiliations:** 1 INSERM UMRs 1097, Parc Scientifique de Luminy, Marseille, France; 2 Aix Marseille Université, Marseille, France; 3 Rhumatologie, IML, AP-HM, Hôpital Sainte Marguerite, Marseille, France; 4 Service d'Immunologie Clinique, Hôpitaux universitaires de Strasbourg, Strasbourg, France; 5 UPR CNRS 3572, Strasbourg, France; 6 Institut d’Immunologie Centre Hospitalier Régional et Universitaire de Lille, Lille, France; 7 EA 2686, Université de Lille, Lille, France; 8 Service de Médecine Interne, Centre National de Référence de la Sclérodermie Systémique, Hôpital Claude Huriez, Lille, France; 9 Service de Médecine Interne et Pathologie Vasculaire, Hôpital St Louis, Paris, France; 10 INSERM U697, Hôpital St Louis, Paris, France; 11 Service de Médecine Interne, Hôpital St Antoine, Paris, France; 12 UMR-S 1076 Endothélium, Pathologies Vasculaires et Cibles Thérapeutiques - Faculté de Pharmacie, Marseille, France; 13 AP-HM, Pôle de Médecine Interne, Centre de Compétence PACA Ouest pour la prise en charge des maladies auto-immunes systémiques, Marseille, France; 14 Aix-Marseille Université, Centre d'Immunologie de Marseille-Luminy (CIML), INSERM, CNRS UMR7280, Marseille, France; 15 Service de Médecine Interne, CHU Bretonneau, Tours, France; IMAGINE, FRANCE

## Abstract

In a pilot ProtoArray analysis, we identified 6 proteins out of 9483 recognized by autoantibodies (AAb) from patients with systemic sclerosis (SSc). We further investigated the 6 candidates by ELISA on hundreds of controls and patients, including patients with Systemic Lupus Erythematosus (SLE), known for high sera reactivity and overlapping AAb with SSc. Only 2 of the 6 candidates, *Ephrin type-B receptor 2 (EphB2)* and *Three prime Histone mRNA EXonuclease 1 (THEX1)*, remained significantly recognized by sera samples from SSc compared to controls (healthy or with rheumatic diseases) with, respectively, 34% versus 14% (*P* = 2.10^−4^) and 60% versus 28% (*P* = 3.10^−8^). Above all, EphB2 and THEX1 revealed to be mainly recognized by SLE sera samples with respectively 56%, (*P* = 2.10^−10^) and 82% (*P* = 5.10^−13^). As anti-EphB2 and anti-THEX1 AAb were found in both diseases, an epitope mapping was realized on each protein to refine SSc and SLE diagnosis. A 15-mer peptide from EphB2 allowed to identify 35% of SLE sera samples (N = 48) versus only 5% of any other sera samples (N = 157), including SSc sera samples. AAb titers were significantly higher in SLE sera (*P*<0.0001) and correlated with disease activity (p<0.02). We could not find an epitope on EphB2 protein for SSc neither on THEX1 for SSc or SLE. We showed that patients with SSc or SLE have AAb against EphB2, a protein involved in angiogenesis, and THEX1, a 3’-5’ exoribonuclease involved in histone mRNA degradation. We have further identified a peptide from EphB2 as a specific and sensitive tool for SLE diagnosis.

## Introduction

Systemic sclerosis (SSc) is a rare severe chronic autoimmune disease beginning with vascular damage, causing collagen synthesis dysfunction and fibrotic phenotype in the skin and internal organs [1]. It is different from localized scleroderma (LocSc) or morphea which classically presents benign and self-limited evolution and is confined to the skin and/or underlying tissues. Systemic sclerosis remains difficult to diagnose because of clinical heterogeneity. Two main forms of SSc have been defined: *limited cutaneous disease (lcSSc)* characterized by skin involvement below the elbows and knees and *diffuse cutaneous disease (dcSSc)* for which skin involvement is more extended. More than 80% of patients with SSc have anti-nuclear antibodies (ANA). Some specific autoantibodies correlate with clinical subtypes and are helpful for diagnosis and clinical classification: anti-centromere antibodies (ACA or anti-CENP-B) and anti-topoisomerase antibodies (ATA or anti-Scl-70) are respectively markers of lcSSc and dcSSc present in 65% and 40% of patients in each clinical subgroup [2]. Additionally the new Classification Criteria for SSc includes the anti-RNA polymerase III antibodies [3], associated with the diffuse cutaneous form of SSc and renal crisis [4, 5], although only present in 6–9% of French patients with SSc [6]. Other biomarkers can be used for SSc classification, although less frequent and less frequently used in routine: anti-fibrilllin (AFA), anti-Th/To, anti-Pm/Scl or anti-U3RNP are associated with particular clinical manifestations [7–10].

Nevertheless about one third of patients with SSc have none of the above mentioned SSc-specific autoantibodies in their sera and a need for new biomarkers is obvious in a disease still difficult to be diagnosed or classified.

Similarly, patients with Systemic Lupus Erythematosus (SLE) have IgG autoantibodies against more than a hundred different antigens including DNA, nucleosomes, histones, viral antigens, transcription factors, only few are useful for the diagnosis of SLE [8]. Patients with SLE are known for high sera reactivity and for having overlapping AAb with SSc.

Anti-dsDNA antibodies help to monitor disease activity and anti-Smith (anti-Sm) antibodies are highly specific for SLE diagnosis [9]. Still, 20 to 30% of patients with SLE do not have anti-dsDNA antibodies and 60 to 80% do not have anti-Sm antibodies. A specific marker for SLE and particularly for disease activity would therefore be useful to adjust treatments.

To identify new autoantibodies in SSc, we first screened 9483 human proteins spotted on protein arrays with 20 sera from patients with SSc, including patients positive for ACA (ACA^pos^), positive for ATA (ATA^pos^) or negative for both antibodies (ATA/ACA^neg^) and 18 sera from controls (healthy and with other autoimmune diseases (AID)). Six proteins were recognized by autoantibodies from half of the patients with SSc and none of the controls: *Fibroblast Growth Factor 2* (FGF2), *Allograft Inflammatory Factor 1* (AIF1), *Ephrin Type-B receptor 2* (EphB2), *Dual specificity protein kinase CLK1*, *Three prime Histone mRNA EXonuclease 1* (THEX1) and *Ankyrin repeat and Sterile alpha motif domain containing 6* (ANKS6). The same six human proteins as those spotted on protein arrays were separately purchased and coated in 96 well plates to be tested by ELISA and further challenged on a large number of patients and controls.

By ELISA, only Ephrin type-B receptor 2 (EphB2) and Three prime Histone mRNA EXonuclease 1 (THEX1) remained significantly recognized by autoantibodies from patients with SSc and were further tested to reach a total of respectively 336 and 362 individuals including patients with SSc, with Systemic Lupus Erythematosus (SLE) Rheumatoid Arthritis (RA), Psoriatic Arthritis (PsA), Ankylosing Spondylitis (AS) and healthy controls (HC).

## Patients and Methods

### Criteria for patients and controls

Patients with Systemic scleroderma (SSc) fulfilled the criteria of LeRoy for SSc [11], while patients with localized scleroderma (LocSc or morphea) were distinguished according to the international classification [12]. Patients with RA satisfied the 2010 revised criteria of the American College of Rheumatology (ACR) and the European League Against Rheumatism (EULAR) [13]. Patients with SLE fulfilled the American College of Rheumatology revised criteria for SLE [14] as updated in 1997 [15]. Patients with Psoriatic Arthritis (PsA) fulfilled the ClASsification of Psoriatic ARthritis (CASPAR) criteria [16, 17] and patients with Ankylosing Spondylitis (AS) fulfilled the Assessment of SpondyloArthritis international Society classification criteria [18].

Healthy controls had no history of autoimmunity and were recruited at the Centre d’Examen de Santé de l’Assurance Maladie (CESAM), Marseille, France. Patients with SSc were recruited at Claude Huriez Hospital, Lille; Nord and La Conception Hospitals, Marseille; St Louis and St Antoine Hospitals, Paris. Patients with RA, PsA and AS were recruited in the Rheumatology Unit of St Marguerite Hospital in Marseille. Patients with SLE were recruited at Hôpitaux Universitaires, Strasbourg; La Conception Hospital, Marseille and CHU Bretonneau, Tours.

### Ethics statements

All participants signed informed consent according to the Declaration of Helsinki [19]. The study is registered at the INSERM under the Biomedical Research Protocol number RBM-04-10 and received the approval of the “Comité de Protection des Personnes de Marseille II” or as a collection registered under the number DC-2008-327.

### Participants’ characteristics for ProtoArray analysis

For ProtoArray analysis, sera samples from 20 patients with SSc, including 8 patients negative for ACA and ATA (ACA/ATA^neg^), 6 positive for ATA and 6 positive for ACA, were compared to 18 controls. Controls included 8 healthy individuals with no history of autoimmune diseases (AID) and 10 patients with other AID including 7 Rheumatoid Arthritis (RA), 1 Systemic Erythematous Lupus (SLE) and 2 Localized Scleroderma (LocSc), the latter being tested to distinguish their profile from systemic sclerosis. The patients’ autoantibody profile (ATA, ACA, ACA/ATA^neg^) and patients’ disease subtype (Lc-SSc, Dc-SSc) was obtained by reviewing medical records. ACA/ATA^neg^ patients are negative for ATA *and* ACA but could be positive for other autoantibodies (anti-RNA polymerase III, anti-U3RNP…).

### Participants’ characteristics for ELISA analyses

After protein array determination of 6 proteins specifically recognized by patients with SSc, ELISA analyses of the six candidates were realized on the same individuals than protoarrays and further tested on a larger cohort of patients and controls recruited from the same hospitals and Centers as described above. Two proteins remained significantly more recognized by sera from patients with SSc than from other controls. Clinical characteristics of patients with SSc, with SLE, with RA, with AS, with PsA and healthy controls tested by ELISA for both proteins are detailed in S1 Table.

### Detection of autoantibodies by protein arrays

Human protein microarrays V5.0 (Invitrogen, Carlsbad, CA, USA) were spotted in duplicate on a nitrocellulose-coated glass slide with 9483 human proteins expressed using a baculovirus expression system, purified from insect cells (See www.thermofisher.com for protein content list 5.0). Arrays were first blocked to avoid non-specific hybridization with Blocking Buffer (1% BSA, 1X PBS, 0.1%Tween^®^ 20) at 4°C for 1 hour (PartnerChip, Evry, France). Sera samples, diluted 1:500 in Probe Buffer (1X PBS, 5 mM MgCl2, 0.5 mM DTT, 5% glycerol, 0.05% Triton^®^ X-100, 1% BSA) were added to arrays and incubated for 90 minutes at 4°C in an incubation/hybridization chamber. Arrays were then washed 3 times for 8 minutes with 20 ml Probe Buffer, before adding a 1.0 μg/ml solution of anti-human IgG conjugated to Alexa Fluor^®^ 647 (Invitrogen, Carlsbad, CA, USA) for 90 minutes at 4°C. Arrays were washed again 3 times as described above and dried at room temperature. Arrays were scanned with a NimbleGen MS 200 scanner (Roche, Basel, Switzerland). Fluorescence data was acquired with GenePix Pro Software and processed using Protoarray Prospector 5.2 (Invitrogen, Carlsbad, CA, USA). Two negative control slides were treated in an identical manner to the experimental assays, except that they were incubated with buffer instead of sera prior to incubation with the Alexa Fluor^®^647-anti-human IgG detection reagent. The two control slides allowed the exclusion of 58 non-specific proteins from the 9483 spotted proteins (see S2 Table).

### ProtoArray^®^ data analysis

ProtoArray^®^ microarrays allowed to identify human proteins that are recognized by specific IgG autoantibodies present in different serum samples by probing with a second Ig-class specific antibody labeled with a fluorescent probe such as Alexa Fluor^®^ 647. Binding of the secondary antibody on the microarray was then quantified by measuring the fluorescence intensity of each feature on the slide. The ProtoArray^®^ Prospector software includes a linear normalization algorithm that facilitates inter-assay data analysis and M-statistics algorithms for cross-group comparisons important for biomarker identification. This M-statistic (Cf. Immune Response Biomarker Profiling Toolbox v5.2, Invitrogen) is used to determine number of patients among SSc group that have a signal value for a probe greater than the highest observed signal value of this probe in the comparison control group (healthy individuals and patients with other AID). This gives a Fluorescence Intensity (FI) cut-off for each protein. Any sera sample superior to this FI cut-off is determined as being positive for autoantibodies against that protein.

### Detection of autoantibodies by ELISA

Proteins specifically recognized by ProtoArray method were purchased from Invitrogen, except Fibroblast Growth Factor 2 (FGF2) which was purchased from Millipore (CA, USA). They were identical to the proteins coated on protoarrays for post translational modifications. 96-well plates (Nunc, Kamstrupvej, Denmark) were coated overnight at 4°C with candidate proteins diluted in PBS. The same patients with SSc who were positive by protoarrays for anti-FGF2, AIF1 EphB2, CLK1 THEX1 or ANKS6 autoantibodies were first validated by ELISA. Thus, working conditions for autoantibody detection were defined at 0.2 μg/well for all proteins except ANKS6 for which 0.1 μg/well was sufficient to detect positive samples. Plates were blocked with PBS 2% BSA overnight. After blocking solution removal, sera samples diluted at 1:100 in PBS 1% BSA were added. After 2 hours of incubation at room temperature, plates were washed 3 times (1–2 minute) with PBS 0.1% Tween 20 and then peroxidase-conjugated anti-human IgG (Sigma Aldrich, St Quentin-Fallavier, France) was added for 30 minutes before being revealed with tetramethylbenzidine (TMB) liquid substrate system (Sigma-Aldrich, St Louis, MO, USA). Absorbance (Abs) was read at 405 nm on a PowerWave XS microplate spectrophotometer (Biotek, Colmar, France). For each individual, background Abs was obtained by adding sera on duplicated wells without tested protein. Positive sera were defined by an Abs value superior or equal to twice the background Abs (positive ΔAbs = 0 or more).

### Epitope mapping on EphB2 and THEX1 protein

In order to determine which part of the protein was recognized by autoantibodies, we performed an epitope mapping for EphB2 and THEX1. A total of thirty-four 15-mer peptides encompassing residues from EphB2 (locus NM_004442.3) overlapping on 7 or 8 amino acids and sixteen 20-mer peptides encompassing residues from THEX1 (locus NM_153332.2) overlapping on 10 amino acids were synthesized using the solid-phase system, and then purified (Polypeptide Laboratories, Strasbourg, France). Plates were coated overnight with 10 μg/well peptides diluted in PBS, pH 7.4 as previously described [20]. Plates were blocked washed and revealed similarly to the ELISA plates presented above. Sera, diluted to 1:100 in PBS 1% BSA, were incubated for 2 h. Positive wells were defined as above (ΔAbs = 0 or more).

### Statistical analysis

To determine whether a candidate protein was significantly better recognized by autoantibodies from patients with SSc or patients with SLE rather than healthy controls and/or patients with other rheumatic diseases, p values were calculated using the χ^2^ test and corrected for multiple comparisons (Bonferroni) depending on the number of groups tested (i.e. when 3 groups were compared—SSc, HC and other rheumatic diseases (RD)—a correction by 3 was applied).

For ΔAbs comparisons between groups, p values were evaluated using Mann Whitney test. Correlation between ΔAbs and severity of the disease (SLEDAI) for SLE was assessed by Spearman’s rank test (Graphpad Prism 6).

To evaluate diagnostic ability of each tested protein for SSc and SLE, plots of sensitivity versus 1-Specifity or Receiver Operating Characteristic (ROC) curves have been realized and the area under the curve (AUC) is given for each protein and disease (Graphpad Prism 6).

## Results

### Identifying new autoantibodies for SSc diagnosis by ProtoArray^®^ analysis

By Protoarrays, we aimed at identifying circulating antibodies that react with specific proteins and were associated with SSc. To validate highly specific and new candidates for SSc diagnosis with an unbiased method, we arbitrarily selected human proteins, among the 9483 present on the chips, which were recognized by sera from at least 50% of the 20 patients with SSc tested and never recognized by sera of the 18 controls (including patients with AID and healthy controls). Only six protein candidates (Table 1) fulfilled this stringent selection (*Patent PCT/EP2013/065490)*: Fibroblast Growth Factor 2 (FGF2), Allograft Inflammatory Factor 1 (AIF1) transcript variant 1, Ephrin Type-B receptor 2 (EphB2), Dual specificity protein kinase CLK1, Three prime Histone mRNA EXonuclease 1 (THEX1), Ankyrin repeat and Sterile alpha motif domain containing 6 (ANKS6).

**Table 1:** Protoarray results of candidate proteins.

			Protein abbreviation		FGF2	AIF1	EphB2	CLK1	THEX1	ANKS6
			*FI cut-off* ^a^		*>605*	*>1 798*	*>1 109*	*>1 535*	*>9 655*	*>567*
ID #	Disease^b^	Disease duration (year)	AutoAb^c^	Sex^d^						
1	Dc-SSc	5.1	ACA/ATA^neg^	F	99	985	352	593	**15 695**	**620**
2	Lc-SSc	1.5	ACA/ATA^neg^	F	**847**	**3 151**	723	651	7 530	559
3	Dc-SSc	5.1	ACA/ATA^neg^	F	473	1 309	542	392	**10 180**	**608**
4	Dc-SSc	10.8	ACA/ATA^neg^	F	57	616	187	280	8 942	**634**
5	Dc-SSc	18.9	ACA/ATA^neg^	F	66	**2 615**	1 092	**3 707**	**15 686**	322
6	Dc-SSc	4.3	ACA/ATA^neg^	F	65	1 588	228	295	**12 080**	400
7	Dc-SSc	0.3	ACA/ATA^neg^	F	21	**3 934**	401	193	**9 845**	**715**
8	Lc-SSc	10.0	ACA/ATA^neg^	F	**18 456**	**1 816**	**3 759**	**8 590**	3 568	273
9	Dc-SSc	7.9	ATA	F	**25 608**	848	**2 679**	**8 520**	3 724	**574**
10	Dc-SSc	1.2	ATA	F	35	**2 394**	417	510	6 745	509
11	Dc-SSc	1.4	ATA	F	**12 522**	**1 927**	**2 163**	**4 306**	**14 205**	365
12	Dc-SSc	5.4	ATA	F	**9 517**	**2 435**	**1 883**	**3 802**	6 325	481
13	Lc-SSc	2.8	ATA	M	**12 700**	446	**3 168**	**5 556**	1 113	222
14	Dc-SSc	0.0	ATA	F	**12 973**	734	**2 152**	**4 711**	**20 297**	**671**
15	Lc-SSc	23.3	ACA	F	**15 007**	**3 438**	**3 233**	**7 466**	**21 136**	**685**
16	Lc-SSc	28.4	ACA	F	**9 637**	388	**1 489**	**3 015**	6 955	**698**
17	Lc-SSc	10.0	ACA	F	**758**	750	**1 338**	261	**22 825**	**716**
18	Lc-SSc	6.8	ACA	F	357	709	418	288	3 705	**621**
19	Lc-SSc	12.9	ACA	F	75	**2 028**	387	1 198	**34 959**	341
20	Dc-SSc	1.2	ACA	F	**10 657**	**4 945**	**1 658**	**6 551**	5 220	328
**Number of SSc patients (N = 20) positive for anti-protein Ab**					**11**	**10**	**10**	**10**	**10**	**10**
**mean FI SSc / FI cut-off**					**10.7**	**1.0**	**1.3**	**2.0**	**1.2**	**0.9**
21	RA	6.0	ACPA	F	52	932	207	468	6 300	319
22	RA	4.0	ACPA	F	39	744	471	621	9 455	560
23	RA	4.0	ACPA	M	17	1 598	292	418	4 022	437
24	RA	25.0	ACPA	F	57	535	315	307	8 723	117
25	RA	21.0	ACPA	F	28	328	197	268	2 782	296
26	RA	3.0	ACPA	F	37	1 117	430	176	4 074	465
27	RA	2.0	ACPA	F	97	1 412	421	231	3 799	444
28	LocSc	5	unk	M	49	547	209	278	4 783	367
29	LocSc	8.7	ACA/ATA^neg^	M	65	998	472	1 335	4 256	475
30	SLE	24	DNA^neg^ Sm^neg^	F	24	1 442	245	162	2 838	254
31	HC	NA	NA	F	16	1 115	126	181	1 563	208
32	HC	NA	NA	F	277	1 042	909	284	5 393	520
33	HC	NA	NA	F	81	1 223	269	387	3 133	275
34	HC	NA	NA	F	52	1 500	582	181	3 349	339
35	HC	NA	NA	F	21	800	144	109	2 414	242
36	HC	NA	NA	F	40	920	195	260	3 335	334
37	HC	NA	NA	F	405	416	368	327	6 639	503
38	HC	NA	NA	F	77	409	197	428	4 461	418
**Number of controls (N = 18) positive for anti-protein Ab**					**0**	**0**	**0**	**0**	**0**	**0**
***P values***^***e***^			***χ*** ^***2***^		*<2*.*10*^*−4*^	*<5*.*10*^*−4*^	*<5*.*10*^*−4*^	*<5*.*10*^*−4*^	*<5*.*10*^*−4*^	*<5*.*10*^*−4*^
			***Mann Whitney***		*<0*.*0001*	*0*.*0353*	*0*.*0003*	*0*.*0003*	*0*.*0006*	*0*.*0043*

^a^FI cut-off: Fluorescence intensity cut-off for determining a positive signal (see ProtoArray^®^ data analysis in section Patients and Methods);

^b^ type of disease: Dc-SSc, Lc-SSc: respectively Diffuse or Limited cutaneous scleroderma; RA: rheumatoid arthritis, LocSc: localized scleroderma (by opposition to systemic), SLE: Systemic lupus erythematosus, HC: healthy controls;

^c^AutoAb status: Autoantibody status: ACA: anti-centromere antibodies, ATA: anti-topoisomerase antibodies, ACA/ATA^neg^: negative for ACA and ATA, ACPA anti-citrullinated protein antibody, anti-Sm: anti-Smith antibodies, UKN: unknown; NA: not applicable;

^d^ sex^:^ F: female, M: male.

P values were calculated using the χ^2^ test for comparisons between positive and negative individuals between groups, or Mann Whitney test for differences in intensity values between groups.

### Anti-EphB2 and anti-THEX1 autoantibodies in Scleroderma and Lupus

The six proteins were separately purchased and tested on a larger number of sera from patients and controls for the presence of AAb. To set-up ELISA working conditions, we first tested patients who had been positive for anti-FGF2, -AIF1, -EphB2, -CLK1, -THEX1 or -ANKS6 AAb by Protoarrays and defined most of them being positive by ELISA at sera dilution 1:100 and at protein concentration of 0.2μg/well, except ANKS6 for which 0.1μg/well was sufficient to detect positive samples.

**Fig 1:**
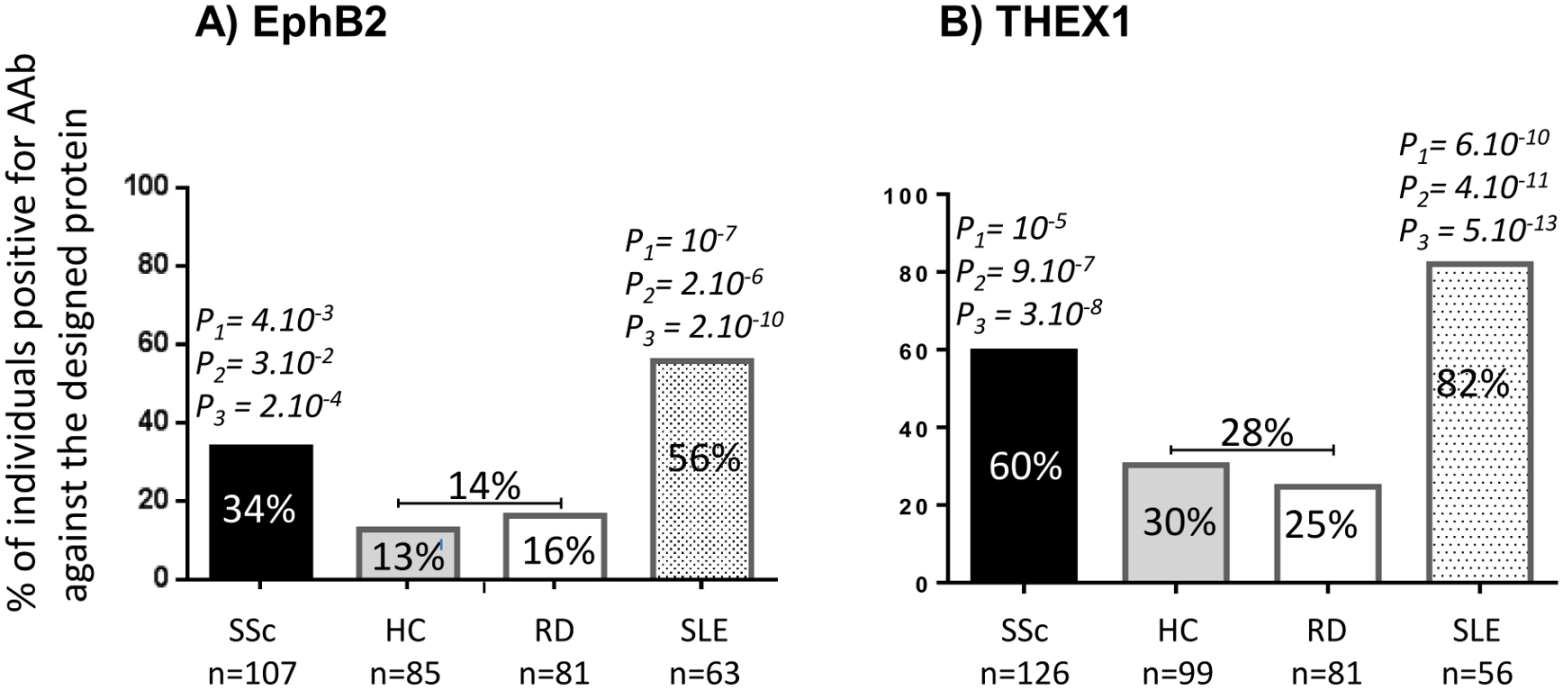
Patients with scleroderma and patients with lupus have autoantibodies (AAb) against A) EphB2 and B) THEX1 proteins. Under our ELISA conditions, both proteins are significantly recognized by sera from patients with systemic sclerosis (SSc) and patients with Systemic Lupus Erythematosus (SLE), when compared to both sera from healthy controls (HC) and sera from patients with other rheumatic diseases (RD). All p values (P) are calculated after Bonferroni correction. P_1_: patients with SSc or SLE compared to HC, P_2_: compared to RD, P_3_: compared to both HC and RD.

Only EphB2 and THEX1 remained significantly recognized by respectively 34% and 60% of sera samples from patients with SSc compared to 14% and 28% (*P* = 2.10^−4^, *P* = 3.10^−8^) of sera samples from all controls including sera samples from healthy controls and from patients with other rheumatic diseases (Fig 1).

Patients with SLE, known for high sera reactivity and overlapping AAb with SSc, were tested in parallel for both proteins and showed a higher reactivity against EphB2 and THEX1 than SSc sera. EphB2 and THEX1 were recognized by respectively 56% and 82% of sera samples from patients with SLE compared to 14% and 28% of other controls (*P* = 2.10^−10^ and *P* = 5.10^−13^).

### Clinical and serological characteristics of patients with SSc having anti-EphB2 or anti-THEX1 autoantibodies

We observe in our study that men with SSc had a tendency to have more often anti-EphB2 AAbs than women with SSc, although without reaching significance (Table 2, *P* = 0.053). Otherwise patients with SSc were not significantly different in age at diagnosis, had similar disease duration and did not differ in ethnicity or clinical subtypes (diffuse or limited SSc), whether they were positive or negative for anti-EphB2 or anti-THEX1 AAb. Patients had similar organ involvement at the time of their blood draw whether they were positive or negative for both AAb except for patients negative for EphB2 AAb for whom joint involvement was marginally more frequent (*P* = 0.04). Treatments were similar in any groups (data not shown).

**Table 2:** Clinical and serological characteristics of patients with SSc either positive or negative for anti-EphB2 or -THEX1 antibodies.

	Anti-EphB2 AAb (n = 107^a^)		Anti-THEX1 AAb (n = 126)	
Sera samples from patients with SSc	Positive (n = 36)	Negative (n = 71)	Positive (n = 75)	Negative (n = 51)
**Female (%)**	72*	87	81	78
**Mean age at diagnosis (years)**	45	46	47	46
**Disease duration (years)**	7.1	6.5	7.3	5.8
**Ethnic diversity (%)**				
** Caucasian**	83	80	75	86
** Asian**	14	7	7	4
** African**	0	14	16	8
**Disease’s subtype**^b^ **(%)**				
** Lc-SSc**	53	51	47	57
** Dc-SSc**	47	49	51	43
**Organ involvement (%)**				
** Lung**	73 (n = 15)	67 *(n = 39)*	67 *(n = 36)*	61 *(n = 36)*
** Cardio-vascular**	33 (n = 12)	36 *(n = 31)*	21 *(n = 28)*	25 *(n = 24)*
** Joint**	42 (n = 12)	76** ***(n = 25)***	63 *(n = 27)*	63 *(n = 27)*

^a^ Number of individuals considered for calculation is indicated ahead of each column, otherwise indicated between brackets (when clinical data are not available for all individuals).

^b^ Patients with SSc are divided into 2 clinical subtypes: limited cutaneous and diffuse cutaneous SSc, respectively, Lc-SSc and Dc-SSc.

*p value = 0.053 (χ^2^ test).

** p value = 0.04 (χ^2^ test).

**Fig 2:**
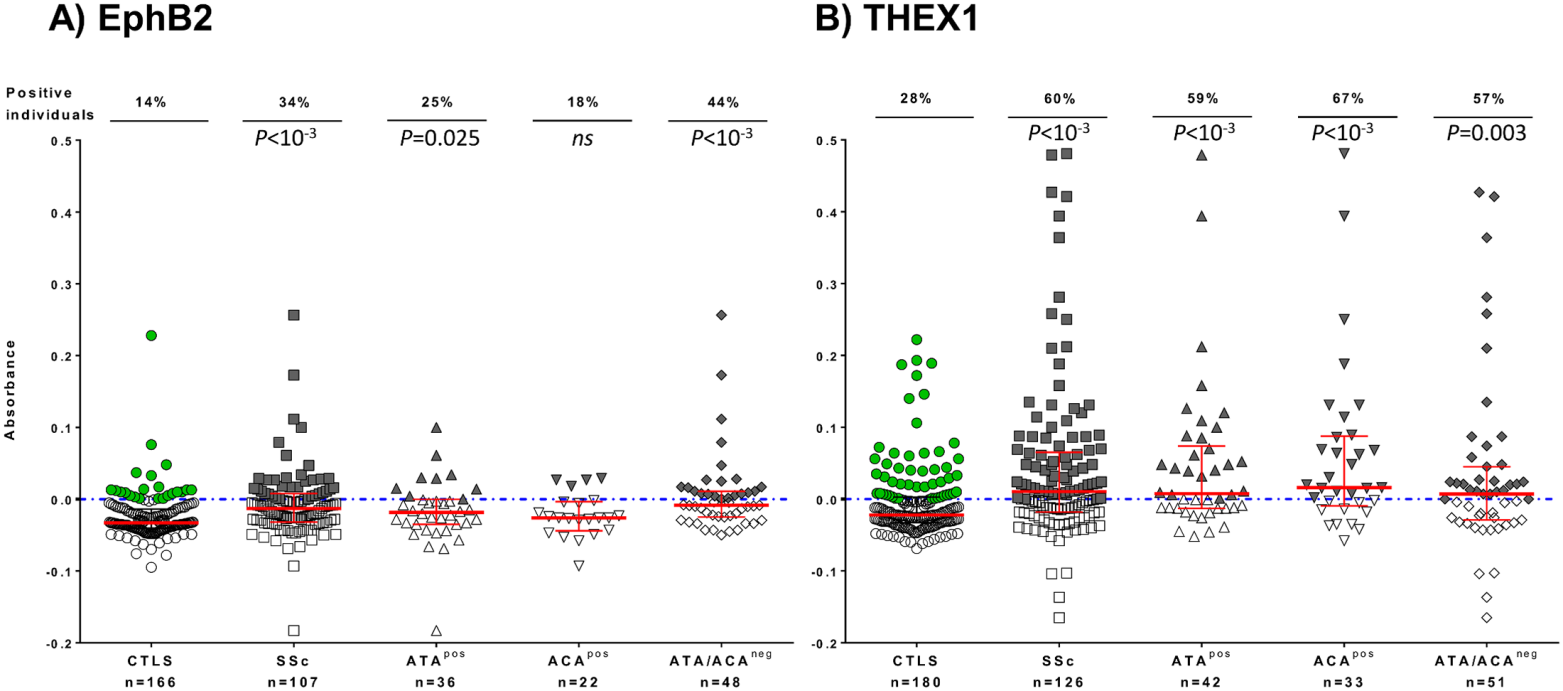
Autoantibodies against A) EphB2 and B) THEX1 analyzed in patients with scleroderma. Sera reactivity against A) EphB2 or B) THEX1 is given by Absorbance (Abs) value for patients with systemic sclerosis (SSc), patients with SSc positive for anti-topoisomerase antibody (ATA^pos^), positive for anti-centromere antibody (ACA^pos^) or negative for both (ACA/ATA^neg^), and compared to all controls (CTLS) including healthy controls (HC) and controls with other Rheumatic diseases (RD), with a total of n = 166 with 85 HC and 81 RD for EphB2 and n = 180 with 99 HC and 81 RD for THEX1. Sera were tested at dilution 1/100 and defined as positive when Abs≥0 (on or above the dotted blue line). Red bars represent medians with interquartile ranges. Percentage of individuals positive for anti-EphB2 or THEX1 antibodies are indicated in the upper part of the graph. All P values are calculated using Mann Whitney test by comparing Abs results from patients with SSc as a whole group or in SSc subgroups to all controls (CTLS).

Nevertheless, when patients were classified according to classical SSc- specific AAb status (ATA^pos^, ACA^pos^ or ATA/ACA^neg^), those without classical SSc-AAb (ACA/ATA^neg^) were the one who had more often anti-EphB2 AAb (44%) and with the highest Absorbance levels (*P*<0.0001, Fig 2A). On the other hand, anti-THEX1 AAb could similarly diagnose any patients with SSc, whether they had or not classical SSc-AAb (Fig 2B). Other AAb, anti-ds-DNA, -SSA, -SSB, -RNP, -PmScl or anti-Jo1 antibodies did not reveal any particular pattern in one group or the other and were rare enough to allow statistical evaluations (data not shown).

### Clinical and serological characteristics of patients with SLE having anti-EphB2 or anti-THEX1 autoantibodies

Patients with SLE were not significantly different in gender, ethnicity, whether they were positive or negative for anti-EphB2 or anti-THEX1 AAb (Table 3). Patients with anti-EphB2 AAb had more often experienced cardiovascular events than patients negative for this AAb (*P* = 0.015, two-sided Fisher’s exact test), whereas patients with anti-THEX1 AAb had more often an active disease (without reaching significance), were more often positive for anti-dsDNA AAb (*P* = 0.017) and had more often joint involvement (*P* = 0.019).

**Table 3:** Clinical and serological characteristics of patients with SLE, positive or negative for anti-EphB2 or -THEX1 antibodies.

	Anti-EphB2 AAb n = 63 ^a^		Anti-THEX1 AAb n = 56	
Sera samples from patients with SLE	Positive *n = 35*	Negative *n = 28*	Positive *n = 46*	Negative *n = 10*
**Female %**	80	89	89	80
**Disease activity (%)**		*(n = 25)*	*(n = 35)*	*(n = 8)*
** in remission**	57	68	66	87
** active**	43	32	34	13
**Ethnic diversity**				
** Caucasian**	91	100	93	100
** Asian**	2	0	4	0
** African**	6	0	2	0
**AutoAb status**^b^ **(%)**				
** dsDNA**	54	48 *(n = 25)*	62* *(n = 37)*	**13** *(n = 8)*
** Sm**	29 *(n = 14)*	20 *(n = 15)*	19 *(n = 26)*	20 *(n = 5)*
** RNP**	21 *(n = 33*	19 *(n = 26)*	19 *(n = 36)*	20
** SSA**	56 *(n = 16)*	50 *(n = 14)*	35 *(n = 23)*	80 *(n = 5)*
** SSB**	40 *(n = 15)*	23 *(n = 13)*	14 *(n = 22)*	40 *(n = 5)*
**Organ involvement (%)**	*(n = 31)*	*(n = 21)*	*(n = 41)*	*(n = 8)*
** Skin**	23	29	22	25
** Joint**	36	19	61*	**13**
** Kidney**	19	33	12	25
** Cardiovascular**	26*	**0**	20	0
** Hematology**	23	19	12	25
** Brain**	0	0	0	0

^a^ Number of individuals considered for calculation is indicated ahead of each column, otherwise indicated between brackets (when clinical data are not available for all individuals).

^b^ AutoAb status: Autoantibody status: dsDNA, anti-double strand DNA antibodies, Sm: anti-Smith antibodies, RNP: anti-ribonucleoprotein antibodies, SSA, anti-SSA antibodies, SSB, anti-SSB antibodies.

*p value <0.05 with two-sided Fisher’s exact test.

**Fig 3:**
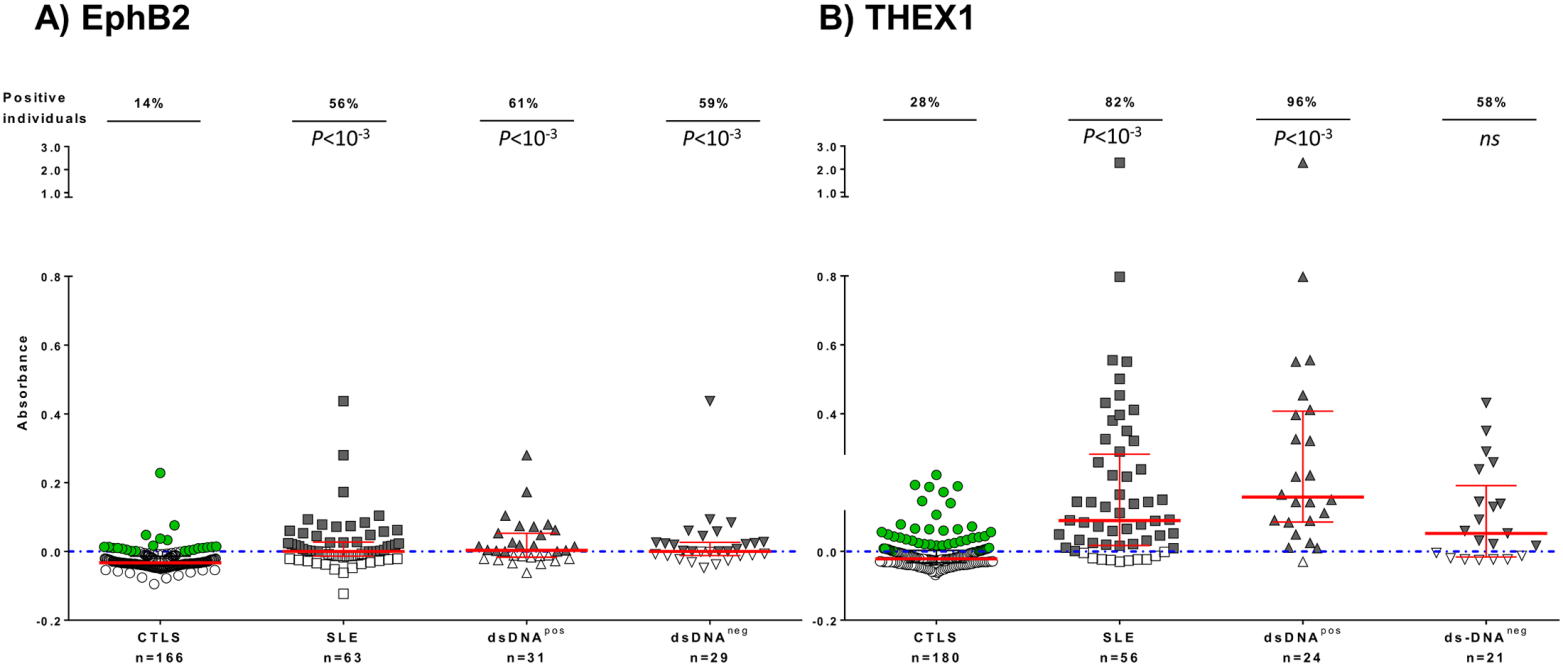
Autoantibodies against A) EphB2 and B) THEX1 analyzed in subgroups of patients with lupus. Sera reactivity against EphB2 or THEX1 is given by Absorbance (Abs) value for patients with Systemic Lupus Erythematosus (SLE), positive for anti-dsDNA antibodies (dsDNA^pos^) or negative (dsDNA^neg^) and compared to all controls (CTLS) including healthy controls (HC) and controls with other Rheumatic diseases (RD), with a total of n = 166 with 85 HC and 81 RD for EphB2 and n = 180 with 99 HC and 81 RD for THEX1. Sera were tested at dilution 1/100 and defined as positive when Abs≥0 (on or above the dotted blue line). Red bars represent medians with interquartile ranges. Percentage of individuals positive for anti-EphB2 or THEX1 antibodies are indicated in the upper part of the graph. All P values are calculated using Mann Whitney test by comparing Abs results from patients with SLE as a whole group or in SLE subgroups to all controls (CTLS).

Anti-EphB2 AAb could diagnose any patient with SLE whether they had or not typical SLE autoantibodies, anti-dsDNA (dsDNA^pos^ or dsDNA^neg^, Fig 3A). Anti-THEX1 AAb preferentially diagnosed SLE patients with anti-dsDNA AAb.

Treatments were similar in any groups (EphB2^pos^ or EphB2^neg^ and THEX1^pos^ or THEX1^neg^); they were often under corticoids or hydroxychloroquine (data not shown).

**Fig 4:**
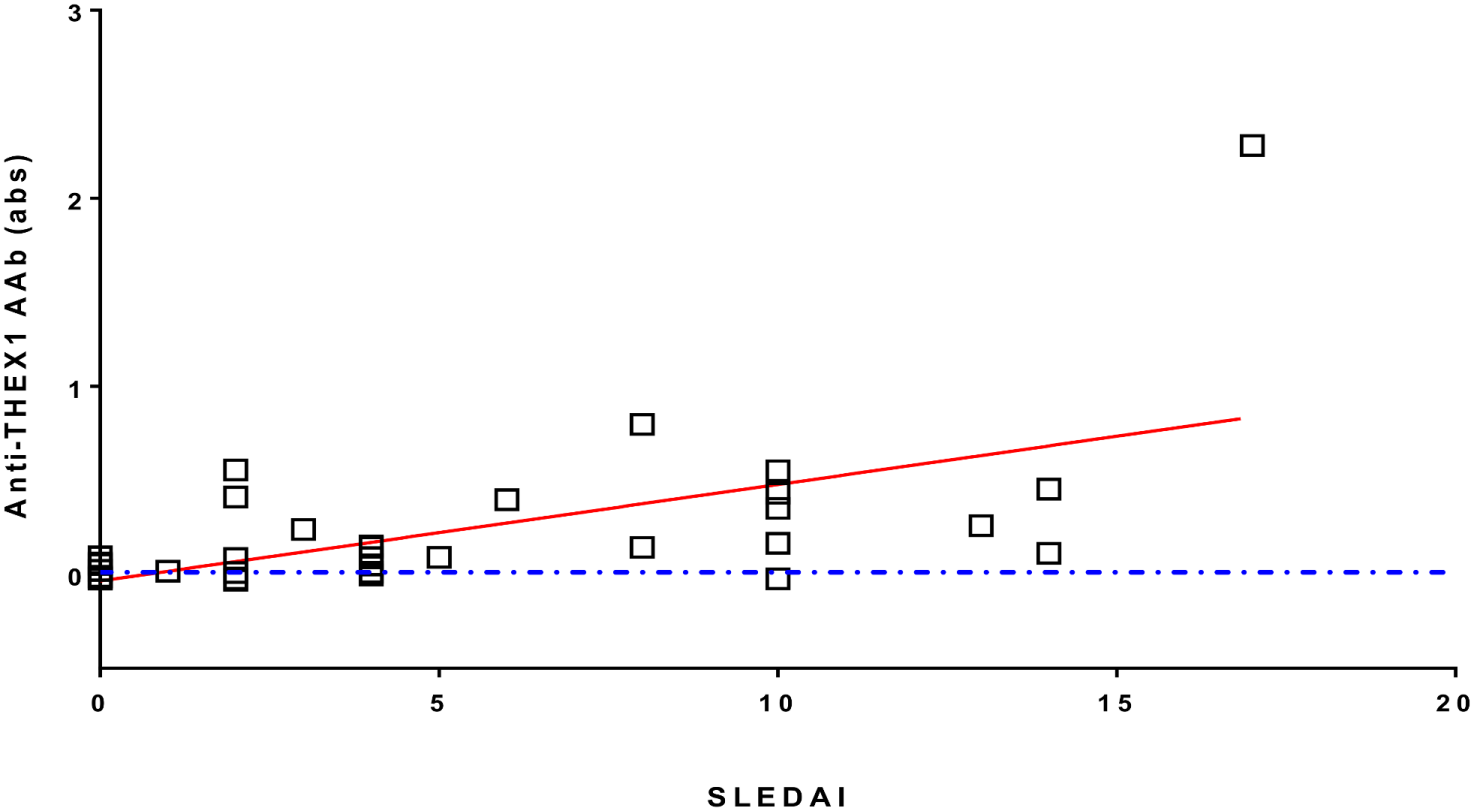
Correlation between anti-THEX1 antibody titers and SLEDAI in patients with SLE. Disease activity is indicated by Systemic Lupus Erythematosus Disease Activity Index (SLEDAI) for 35 patients with SLE. Sera were tested at dilution 1/100 and defined as positive when Abs≥0 (on or above the dotted blue line). Spearman’s correlation r = 0.5843, P (two-tailed) = 0.0002.

Nevertheless in anti-THEX1 AAb detection assays, absorbance levels were higher for patients with SLE (Mean ΔAbs: 0.129, Fig 3B) than for patients with SSc (Mean ΔAbs: 0.01, Fig 2B), suggesting higher AAb titers in the former. Moreover, not only did anti-THEX1 AAb preferentially diagnosed SLE patients with anti-dsDNA AAb, a marker of disease activity, but highest absorbance levels significantly correlated with higher disease activity indexes (Fig 4).

### Comparison of anti-THEX1 and anti-EphB2 AAb for their ability to diagnose SSc and SLE

**Fig 5:**
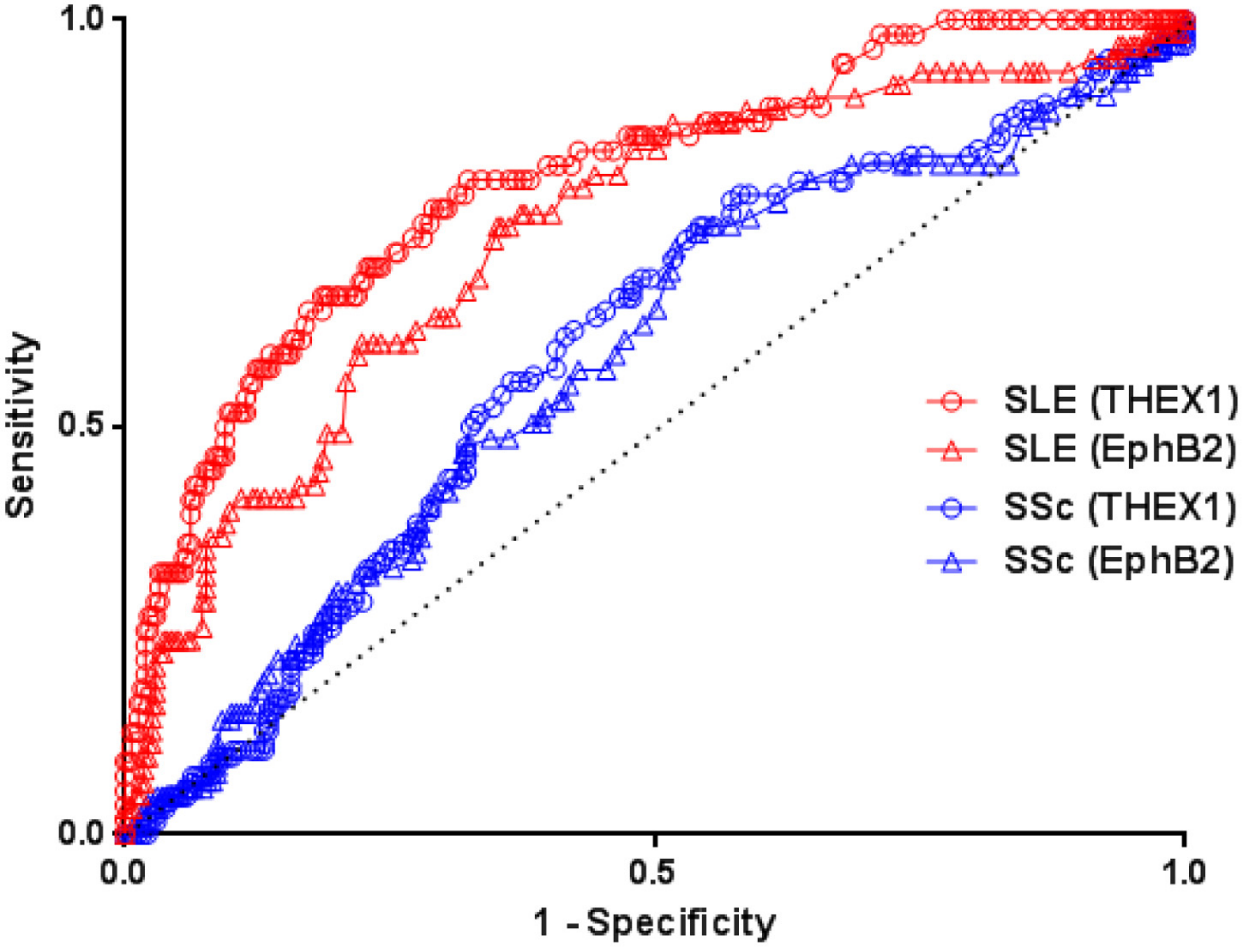
ROC curve analysis with comparison of the ELISA for THEX1 and EphB2 in SSc and SLE. Here are compared diagnostic abilities of EphB2 and THEX1 for SSc and SLE. Controls in both cases are all other individuals, which means patients with SSc were also included among controls when SLE is regarded as the tested disease and inversely patients with SLE were included among controls when SSc is regarded as the tested disease. Areas under the curve (AUC) for THEX1 and EphB2 in SLE are respectively 0,80 and 0,74. AUC for THEX1and EphB2 in SSc are respectively 0,59 and 0,58.

To evaluate diagnostic ability of each tested protein for SSc and SLE, plots of sensitivity versus 1-Specifity or Receiver Operating Characteristic (ROC) curves have been realized by comparing results in each disease to all other subjects. The presence of anti-THEX1 AAb or anti-EphB2 AAb is a good diagnostic tool for SLE even when patients with SSc are also considered as controls with healthy controls and controls with RD (areas under the curve (AUC) of 0.80 and 0.74 respectively). On the opposite, and expected from results above, EphB2 and THEX1 are not diagnostic tools for SSc when among controls patients with SLE are also included (Fig 5).

Anti-THEX1 AAb detection assay allowed a sensitivity of 82% and a specificity of 59% for SLE diagnosis. Anti-EphB2 AAb detection assay allowed a sensitivity of 56% and a specificity of 79% for SLE.

Combination of both proteins (having both anti-THEX1 and anti-EphB2 AAb) allowed to identify 49% (17/35) of patients with SLE, 26% (26/100) of patients with SSc and only 3% (5/153) of all other controls. This gives for SLE diagnosis a lower sensitivity (49%) but a better specificity (88%) than evaluation of individual proteins. For SSc, this allows 26% of sensitivity and 88% of specificity.

### Epitope mapping on EphB2 and THEX1 protein

Although anti-EphB2 and anti-THEX1 autoantibodies were better tools for SLE diagnosis, they were nevertheless found in SSc. We therefore proposed to define disease specific autoantigenic epitopes on both proteins to better refine SSc and SLE diagnosis by screening peptides encompassing residues from EphB2 and THEX1.

**Fig 6:**
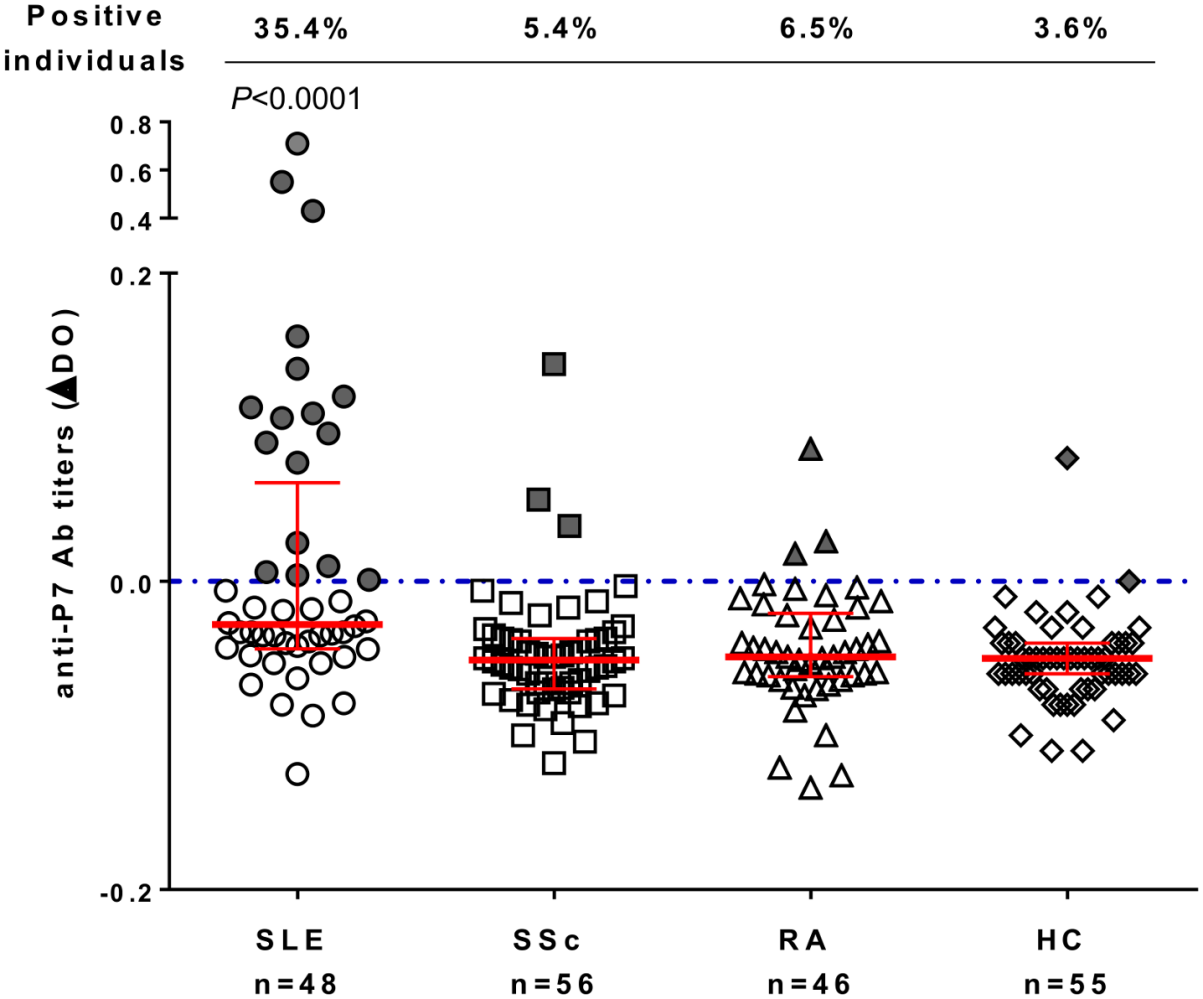
Autoantibodies against peptide 7 (P7 from EphB2 protein) analyzed in patients with scleroderma (SSc), lupus (SLE), Rheumatoid Arthritis (RA) and healthy controls (HC). Sera reactivity against the peptide 7 (P7 at 10μg/well) is given by Absorbance (Abs) values for all patients and controls. Sera were tested at dilution 1/100 and defined as positive when Abs≥0 (on or above the dotted blue line). Red bars represent medians with interquartile ranges. Percentage of individuals positive for anti-P7 antibodies are indicated in the upper part of the graph. One data point for SLE is outside the Y axis limit (-0,41) and not represented here but counted for statistics. P value is calculated using Mann Whitney test by comparing patients with SLE to all controls (SSc, RA and HC, n = 157).

**Fig 7:**
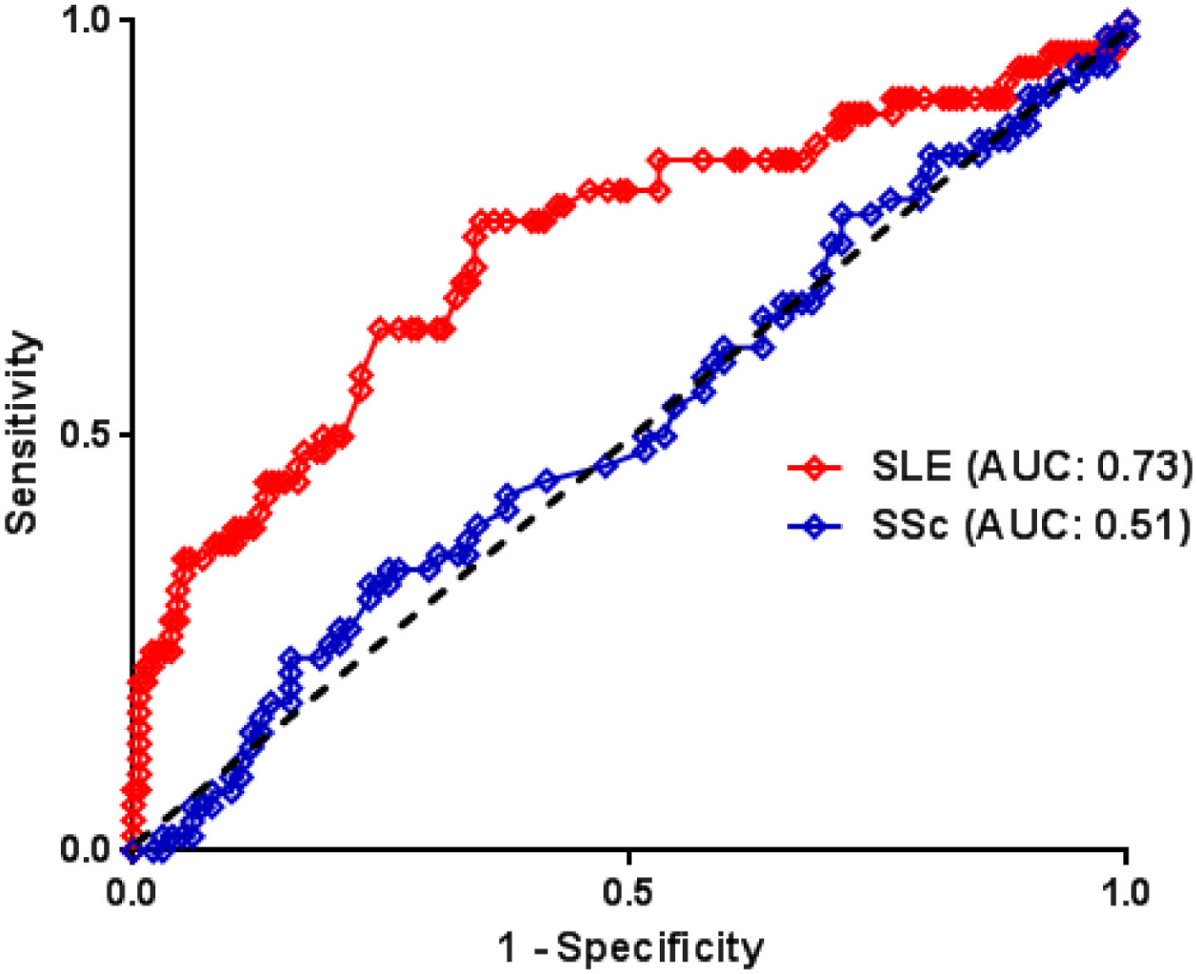
ROC curve analysis for peptide 7 from EphB2 protein (P7). Here are compared diagnostic abilities of P7 for SSc and SLE. Controls in both cases are all other individuals, which means patients with SSc were included among controls when SLE is regarded as the tested disease and inversely patients with SLE were included among controls when SSc is regarded as the tested disease.

Out of the thirty-four 15-mer EphB2 peptides screened, we could find one peptide specifically recognized by sera from patients with SLE: the peptide #7, P7 (Phe-Leu-Ser-Glu-Ala-Ser-Ile-Met-Gly-Gln-Phe-Asp-His-Pro-Asn-NH2). P7 was recognized by 35.4% of patients with SLE (n = 48, P<10^−7^) compared to 5% of all other individuals (N = 157), including 5.4% of patients with SSc (n = 56), 6.5% of patients with RA (n = 46) and 3.6% of healthy controls (n = 55). Sera samples from patients with SLE had titers significantly higher than any other sera (P<0.0001, Fig 6). Roc curve for P7 showed an AUC of 0.73. Anti-P7 AAb detection assay allowed a sensitivity of 35% and a specificity of 95% for SLE diagnosis (Fig 7).

**Fig 8:**
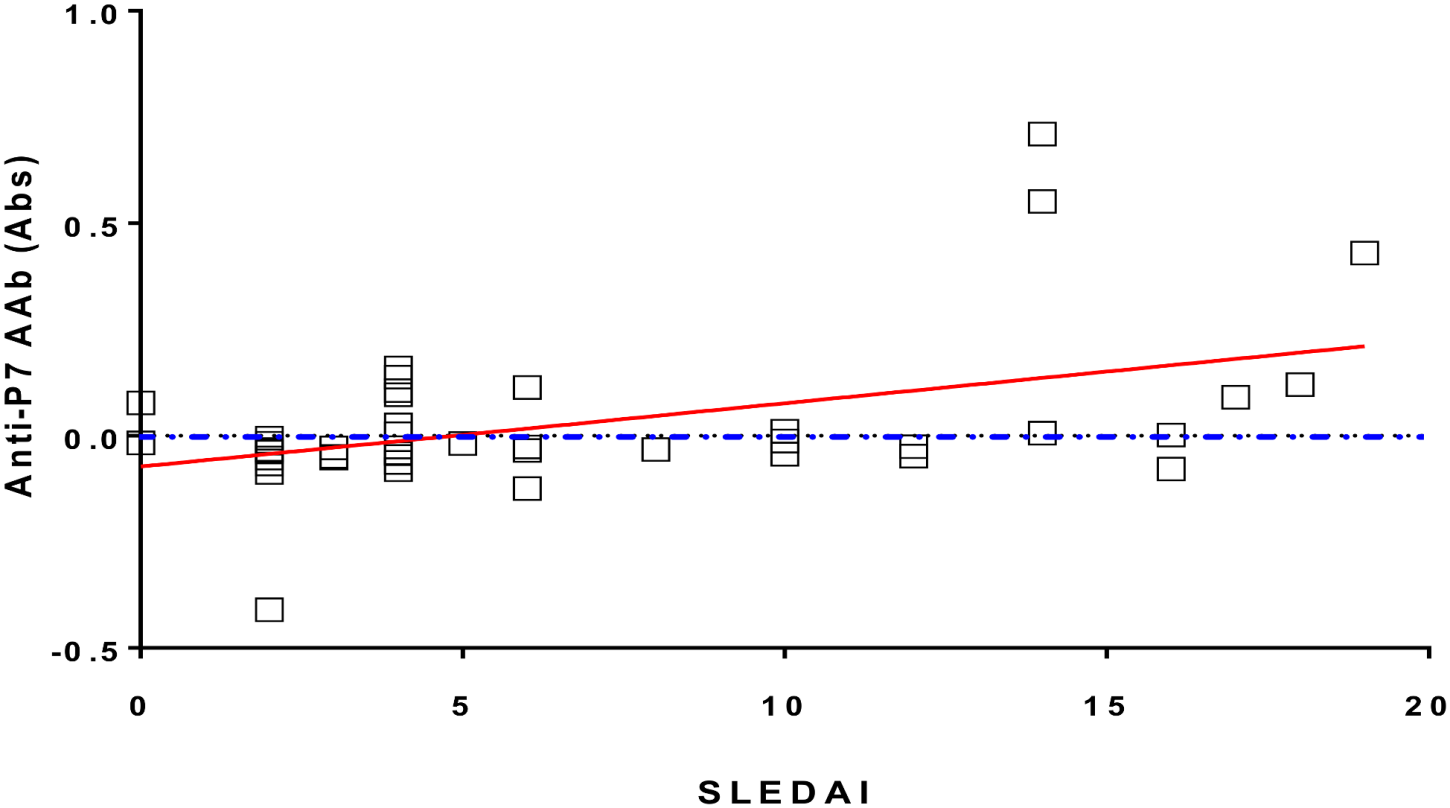
Correlation between anti-P7 antibody titers and SLEDAI in patients with SLE. Disease activity is indicated by Systemic Lupus Erythematosus Disease Activity Index (SLEDAI) for 48 patients with SLE. Sera were tested at dilution 1/100 and defined as positive when Abs≥0 (on or above the dotted blue line). Spearman’s correlation r = 0.3352, P (two-tailed) <0.02.

Interestingly, 30% of sera from patients with SLE without anti-dsDNA-autoantibodies were positive for anti-P7 AAb (data not shown). Notwithstanding, higher anti-P7 antibody absorbances correlated with higher disease indexes and could be a marker of disease activity, although the correlation was marginally significant (Spearman’s rank test, p<0.02, Fig 8).

We could not find an epitope on EphB2 protein specifically recognized by sera from patients with SSc.

THEX1 epitope mapping was similarly realized but we could not find an epitope for SSc or SLE (16 peptides tested, data not shown).

## Discussion

Using ProtoArray^®^ method, we first defined 6 out of 9483 spotted human proteins specifically recognized by IgG autoantibodies from 20 sera of patients with SSc: Fibroblast Growth Factor 2 (FGF2), Allograft Inflammatory Factor 1 transcript variant 1 (AIF1), Ephrin Type-B receptor 2 (EphB2), Dual specificity protein kinase CLK1 (CLK1), Three prime Histone mRNA EXonuclease 1 (THEX1) and Ankyrin repeat and Sterile alpha motif domain containing 6 (ANKS6). Autoantibodies against these proteins have never been described before in SSc, although some of the proteins are involved in SSc pathogenesis. High levels of FGF2, a pro-angiogenic growth factor, are present in plasma from patients with SSc and participate in angiogenesis deregulation in SSc [21]. Overexpression of AIF1, a cytoplasmic inflammation-responsive protein, is observed in affected blood vessels of the lung and skin from patients with SSc, in particular in untreated patients, with early onset of disease [22]. Moreover, several single nucleotide polymorphisms (SNPs) have been described in AIF1 gene and some are associated with SSc [23, 24]. Finally, one of the 3 ligands of EphB2, Ephrin B2, a protein involved in angiogenesis, is up-regulated in clinically involved skin of SSc patients [25]. Therefore, finding autoantibodies against these proteins has a particular signification in the context of scleroderma.

Nevertheless, our protoarray study was driven on a limited number of sera, because of the expensive price of chips, and selected proteins needed to be confirmed for their antigenicity by ELISA on larger number of patients and controls. When tested on more than one hundred sera from patients with SSc, only anti-EphB2 and anti-THEX1 autoantibodies remained statistically more often present in SSc sera compared to other sera from healthy controls or other rheumatic diseases (RA, PsA and AS) in our ELISA conditions.

THEX1, also called ERI-1, is a 3’-5’ exoribonuclease: it binds to the 3’ end of histone mRNAs and degrades them by removing terminal nucleotides from the 3' end [26]. It may play an essential role in histone mRNA decay after replication. It is also able to bind other mRNAs and degrade the 3’-overhangs of short interfering RNAs (siRNAs) and microRNAs [27].

According to our results, THEX1 could be a good diagnostic marker for SSc patients whether they have or not classical SSc autoantibodies (ATA or ACA) as 60% of patients with SSc had AAb in their sera compared to only 28% of healthy controls or controls with RD. Nevertheless when THEX1 was tested for its reactivity on sera samples from patients with SLE, known for having AAb against several proteins including some shared with SSc, THEX1 was a better diagnosis tool for lupus, with 82% of sera recognizing the protein. It could also be a good predictor of flares in SLE as higher anti-THEX1 AAb titers correlated with higher disease activity indexes (*P* = 0.0002). Still, AAb against THEX1 were present in sera from both diseases, we therefore proposed to define disease specific autoantigenic epitopes to better refine SSc and SLE diagnosis by screening peptides encompassing residues from THEX1. Unfortunately, we could not identify a particular epitope for SSc or for SLE on THEX1 protein. This could be due to conformational epitopes that would not be recognized when using linear peptides.

As indicated by its RNA-specific functions THEX1 localizes mostly in the cytoplasm. This suggests that autoantibodies recognizing THEX1, in SSc or SLE, may appear after cell death and exposure of intra-cytoplasmic proteins, or could, as seen for other auto-antibodies [28, 29], penetrate into living cells and migrate to the cytoplasm to bind to cytoplasmic THEX1 proteins. We do not know, and this is beyond the scope of the current study, whether autoantibodies against THEX1 impair its exonuclease function.

Eph receptor tyrosine kinases and their Ephrin ligands represent an important signaling system with widespread roles in cell physiology and disease [30]. Receptors and ligands in this family are anchored to the cell surface and the B family of Eph receptors and Ephrin ligands play essential roles in vascular development and postnatal angiogenesis [30]. EphB receptors and ephrin B ligands are expressed by endothelial cells from various tissues.

EphB2 is a good diagnostic marker for SSc as it is recognized by AAb from 34% of patients compared to only 14% of controls. Moreover 44% of patients who do not have classical SSc-specific autoantibodies (ATA/ACA^neg^) had anti-EphB2 AAb. Nevertheless EphB2 revealed to be better diagnostic tool for SLE as 56% of SLE sera were positive for anti-EphB2 AAb. Moreover, we could spot a 15-mer peptide (P7) on EphB2 protein allowing a 95% specificity for SLE diagnosis with a 35% sensitivity. Anti-P7 antibody titers correlated with disease indexes (SLEDAI) and could be a marker of disease activity. In our conditions, we could not find a SSc-specific epitope on EphB2 protein.

Anti-EphB2 AAb may have a role on vascular functions in both diseases. Furthermore both SLE and SSc are characterized by vascular inflammation, altered angiogenesis, and increased cardiovascular morbidity and mortality. We did not find any difference for cardiovascular involvement at the time of blood draw between patients with SSc positive or negative for anti-EphB2 AAb. This is perhaps due to the fact that all the vascular changes occur in almost all of SSc patients very early in the disease with variable degrees of severity latter. Indeed we had a 10 year follow-up for 11 patients and results indicate that one third of patients have now vascular involvement among patients who were positive for EphB2 AAb and none among patients negative for EphB2 AAb (data not shown). Moreover, we found that men with SSc have a tendency to have more often anti-EphB2 AAbs than women with SSc (p = 0.053). This is an interesting observation, knowing that, although more common in women, SSc appears as strikingly more severe in men and recent results obtained through the EULAR Scleroderma Trials and Research group database demonstrate a higher risk of severe cardiovascular involvement in men [31].

Vasculopathy is also one of the typical symptoms reported in SLE but only in 10 to 40% of patients and may precede the development of a full-blown SLE [32]. Interestingly, we observed that patients with lupus positive for anti-EphB2 AAb had more often experienced cardiovascular events than patients negative for this AAb at the time of blood draw.

Anti-EphB2 autoantibodies are not only good tools for SLE diagnosis, but may have a functional role in vasculopathy. It is to note that AAb recognize the kinase domain of EphB2, an intracytoplasmic region. Again AAb may recognize intracytoplasmic exposed protein structures after cell death or may penetrate living cells and interfere with protein function. This is also behind the scope of this study but future studies would be worth exploring whether anti-EphB2 AAb are capable to penetrate living endothelial cells and dysregulate endothelial cell sprouting. Recent work has reported that EphB2 participate in complex molecular mechanisms that drive endothelial cell movement and the formation of new vessels [33]. If indeed anti-EphB2 AAb disturb the vascular network, this gives also therapeutic perspectives to the peptide P7, or peptides encompassing this epitope, as a tolerogenic peptide hampering AAb to react against their target.

## Conclusion

In conclusion, we showed that patients with SSc or SLE have AAb against EphB2, a protein involved in angiogenesis, and THEX1, a 3’-5’ exoribonuclease involved in histone mRNA degradation. Both proteins are interesting for SSc diagnosis particularly for being recognized by sera from patients without classical SSc autoantibodies (anti-centromere and anti-topoisomerase antibodies). Nevertheless anti-EphB2 and anti-THEX1 AAb are more often and at higher titers present in sera from SLE patients. Moreover, we have further identified a 15-mer peptide from EphB2 protein as a specific and sensitive tool for SLE diagnosis.

## Supporting Information

S1 Table Participants’ characteristics for EphB2 and THEX1 ELISA assays. (DOCX)

S2 Table List of non-specific proteins from the 9483 proteins spotted on human protein arrays. (DOCX)
