# Supplementary material for: Anti-Ephrin Type-B Receptor 2 (EphB2) and Anti-Three Prime Histone mRNA EXonuclease 1 (THEX1) Autoantibodies in Scleroderma and Lupus
Source: PLoS One. 2016 Sep 12;11(9):e0160283. doi: 10.1371/journal.pone.0160283 (PMC5019431; doi:10.1371/journal.pone.0160283)
Supplement: S1 Table — (DOCX) [file pone.0160283.s001.docx]

| **EphB2 assays** | **SSc^a^**  **N=107**^b^ | **SLE**  **N=63** | **RA**  **N=41** | **PsA**  **N=27** | **AS**  **N=13** | **HC**  **N=99** |
| --- | --- | --- | --- | --- | --- | --- |
| Mean age, [range] | 53 [23-78] | 39 [15-84] | 65 [37-90] | 56 [38-85] | 59 [39-84] | 60 [45-77] |
| Sex : female % | 82% | 84% | 76% | 59% | 38% | 100% |
| Positive for autoantibodies^c^, % | ACA 21%  ATA 34%  ACA/ATAneg 45%  (N=106) | dsDNA 53.3% (N=60) | ACPA 100% | NA^d^ | NA | NA |
| Mean age at diagnosis, [range] | 47[12-75] | UKN^e^ | 44 [22-73] | 38 [15-73] | 42 [24-67] | NA |
| Clinical subtype, % | LcSSc,  DcSSc | In remission, 62%  Active disease, 38% (N=60) | NA | NA | NA | NA |
| Caucasian, % | 79% | 95% (N=59) | UKN | UKN | UKN | 98% |
| **THEX1 assays** | **SSc^a^**  **N=126**^b^ | **SLE**  **N=56** | **RA**  **N=41** | **PsA**  **N=27** | **AS**  **N=13** | **HC**  **N=99** |
| Mean age, [range] | 53 [17-85] | 42 [19-84] | 65 [37-90] | 56 [38-85] | 59 [39-84] | 60 [45-77] |
| Sex : female % | 80% | 87% | 76% | 59% | 38% | 100% |
| Positive for autoantibodies^c^, % | ACA 27%  ATA 33%  ACA/ATA^neg^ 36%  (N=125) | dsDNA 52%  (N=44) | ACPA 100% | NA^d^ | NA | NA |
| Mean age at diagnosis, [range] | 46 [12-75] | UKN^e^ | 42 [22-73] | 38 [15-73] | 42 [24-67] | NA |
| Clinical subtype, % | LcSSc, 51%  DcSSc, 49%  (N=124) | In remission, 65%  Active disease, 35%  (N=43) | NA | NA | NA | NA |
| Caucasian, % | 81% | 93%  (N=43) | UKN | UKN | UKN | 98% |

^a^Diseases: SSc, systemic sclerosis ; SLE, systemic lupus erythematosus ; RA, rheumatoid arthritis ; PsoA, psoriatic arthritis ; AS, ankylosing spondylitis ; HC, healthy controls; ^b^ Number of individuals considered for calculation is indicated for each column otherwise indicated between brackets when clinical data are missing; ^c^Autoantibodies: ACA, anti-centromere antibodies ; ATA, anti-topoisomerase antibodies ; ACA/ATA^neg^, without ACA and ATA ; dsDNA, anti-double strand DNA antibodies ; ACPA, anti-citrullinated protein antibodies ; ^d^NA, not applicable, ^e^UKN, unknown.

**S1 Table. Participants’ characteristics for EphB2 and THEX1 ELISA assays.**

| **Database ID** | **Ultimate ORF ID** | **Description** |
| --- | --- | --- |
| BC005332.1 | IOH7177 | cDNA clone MGC:12418 IMAGE:3934658, complete cds |
| BC007782.2 | IOH6514 | immunoglobulin lambda constant 1 (Mcg marker) (IGLC1) |
| BC026038.1 | IOH13982 | Ig gamma-1 chain C region |
| NM_205833.1 | IOH41224 | immunoglobulin superfamily, member 1 (IGSF1), transcript variant 2 |
| NM_032855.1 | IOH14623 | hematopoietic SH2 domain containing (HSH2D) |
| BC032451.1 | IOH21663 | cDNA clone MGC:40426 IMAGE:5178085, complete cds |
| BC056911.1 | IOH29120 | dual specificity phosphatase 15 (DUSP15) |
| BC034142.1 | IOH23177 | immunoglobulin kappa variable 1-5 (IGKV1-5) |
| BC030813.1 | IOH23055 | cDNA clone MGC:22645 IMAGE:4700961, complete cds |
| NM_016207.2 | IOH14059 | cleavage and polyadenylation specific factor 3, 73kDa (CPSF3) |
| BC092518.1 | IOH62695 | Ig gamma-1 chain C region |
| NM_014481.2 | IOH4887 | APEX nuclease (apurinic/apyrimidinic endonuclease) 2 (APEX2), nuclear gene encoding mitochondrial protein |
| BC073791.1 | IOH63073 | immunoglobulin kappa constant, mRNA (cDNA clone MGC:88809 IMAGE:6279986), complete cds |
| BC000468.1 | IOH3618 | ubiquitin-conjugating enzyme E2 variant 1 (UBE2V1) |
| NM_016364.2 | IOH12214 | dual specificity phosphatase 13 (DUSP13), transcript variant 6 |
| BC072419.1 | IOH62555 | Ig gamma-1 chain C region |
| BC090938.1 | IOH62696 | Ig gamma-1 chain C region |
| NM_032017.1 | IOH40615 | serine/threonine kinase 40 (STK40) |
| BC030814.1 | IOH23035 | immunoglobulin kappa variable 1-5 (IGKV1-5) |
| BC056256.1 | IOH29440 | immunoglobulin kappa constant (IGKC) |
| BC002448.2 | IOH4300 | actin binding LIM protein 1 (ABLIM1) |
| BC029444.1 | IOH23178 | immunoglobulin kappa constant (IGKC) |
| BC053984.1 | IOH29361 | immunoglobulin heavy variable 4-31 (IGHV4-31) |
| BC053656.1 | IOH28981 | EGF-like repeats and discoidin I-like domains 3 (EDIL3) |
| BC062732.1 | IOH62856 | Ig kappa chain C region |
| BC070334.1 | IOH40810 | immunoglobulin kappa constant (IGKC) |
| BC022098.1 | IOH14790 | cDNA clone MGC:31944 IMAGE:4878869, complete cds |
| BC034141.1 | IOH23179 | immunoglobulin kappa constant (IGKC) |
| BC020233.1 | IOH14752 | cDNA clone MGC:31936 IMAGE:4765518, complete cds |
| BC025314.1 | IOH13984 | immunoglobulin heavy constant gamma 1 (G1m marker) (IGHG1) |
| BC015833.1 | IOH14840 | cDNA clone MGC:27152 IMAGE:4691630, complete cds |
| NM_003141.2 | IOH9948 | tripartite motif-containing 21 (TRIM21) |
| NM_005030.2 | IOH5070 | polo-like kinase 1 (Drosophila) (PLK1) |
| BC016380.1 | IOH23077 | cDNA clone MGC:27376 IMAGE:4688477, complete cds |
| NM_032349.1 | IOH6316 | nudix (nucleoside diphosphate linked moiety X)-type motif 16-like 1 (NUDT16L1) |
| BC016381.1 | IOH13653 | immunoglobulin heavy constant mu (IGHM) |
| BC034146.1 | IOH23168 | immunoglobulin kappa variable 1-5 (IGKV1-5) |
| BC033178.1 | IOH23236 | immunoglobulin heavy constant gamma 3 (G3m marker) (IGHG3) |
| BC014271.2 | IOH13908 | endoglin (Osler-Rendu-Weber syndrome 1) (ENG) |
| BC002369.1 |  | Serine/threonine-protein kinase PLK1 |
| BC051762.1 | IOH28838 | Uncharacterized protein C20orf96 |
| PHC1705 |  | fms-related tyrosine kinase 3 ligand (FLT3LG) |
| PHR5001 |  | Recombinant human CTLA-4/Fc |
| BC030983.1 | IOH23183 | immunoglobulin lambda locus (IGL@) |
| BC030984.1 | IOH23182 | cDNA clone MGC:32654 IMAGE:4701898, complete cds |
| BC041037.1 | IOH28003 | immunoglobulin heavy constant mu (IGHM) |
| BC014667.1 | IOH14303 | immunoglobulin heavy constant gamma 1 (G1m marker) (IGHG1) |
| BC054893.1 | IOH29495 | immunoglobulin lambda variable 2-14 (IGLV2-14) |
| BC012876.1 | IOH10177 | Ig lambda chain C regions |
| NM_024692.3 | IOH42634 | CAP-GLY domain containing linker protein family, member 4 (CLIP4) |
| BC022362.1 | IOH14191 | cDNA clone MGC:23888 IMAGE:4704496, complete cds |
| BC019337.1 | IOH12297 | immunoglobulin heavy constant gamma 1 (G1m marker) (IGHG1) |
| BC006423.1 |  | Serine/threonine-protein kinase 6 |
| BC017959.1 | IOH12310 | chromosome 2 open reading frame 47 (C2orf47) |
| NM_000159.2 | IOH4104 | glutaryl-Coenzyme A dehydrogenase (GCDH), nuclear gene encoding mitochondrial protein, transcript variant 1 |
| BC006005.1 | IOH7509 | unconventional SNARE in the ER 1 homolog (S. cerevisiae) (USE1) |
| BC053667.1 | IOH29009 | lectin, galactoside-binding, soluble, 3 (LGALS3) |
| BC002755.1 |  | MAP kinase-interacting serine/threonine-protein kinase 1 |

**S1 Table 2. List of non-specific proteins from the 9483 proteins spotted on human protein arrays.**
